# Case of Ozœna and Neuralgia, Cured by the Extraction of a Tooth

**Published:** 1840

**Authors:** Chapin A. Harris

**Affiliations:** Dentist, Baltimore


*Case of Ozæna, accompanied by frequent paroxysms of Neuralgia Faciei, cured by the Extraction of a Tooth.* By Chapin A. Harris, M. D. Dentist, Baltimore.

Mr. S———. a resident of a neighboring county, of a full habit, and slightly disposed to scorbutus, had, for a little more than two years, been subject to an obstinate and distressing affection of the left nasal fossa, and of frequent attacks of pain, which he represented as being at times almost excruciating—commencing immediately over the first left superior molaris, thence shooting back to the angle of the jaw, then to the ala of the nose, inner angle of the eye, and not unfrequently to the top of the head. Ulceration had taken place in the mucous membrane of the affected nostril, and a thin fetid matter, occasionally streaked with pus and blood, was almost constantly discharged, excoriating the parts with which it came in contact. The cavity of the nostril had become so much closed by the thickening of its membranes, that the passage of air through it was prevented; the external integuments had assumed a dark florid appearance, and become considerably tumified and sensitive to the touch.

His teeth having been suspected, though to all appearance perfectly sound, as having some agency in the production of the neuralgic affection, he was directed to a dentist to have them examined, but as none of them exhibited any signs of decay, it was thought to be dependent upon some other cause. Accordingly the remedial means usually employed for this, as well as those for the other affection under which he was laboring, were prescribed; but from their use, although continued for several months, and under a variety of modifications, he derived no benefit.

His complaints becoming more and more aggravated, he at length became apprehensive as to their result, and determined by the advice of his friends, to visit several of the medicinal springs in Virginia. At one of these, he met with an eminent medical gentleman from one of the northern cities, whom he consulted, but neither from his prescription nor the use of the waters of any of the springs that he visited, did he obtain the slightest relief, and after remaining from home two months, he returned in a state almost bordering on despair.

To add to his affliction, he about this time, began to be annoyed with a constant pain in the region of the antrum of the affected side. This, in connection with a soreness in a tooth immediately beneath, which he had felt throughout the whole course of his protracted and complicated disease, but which had not until now been sufficiently great to attract particular observation, soon led to the discovery of the cause both of the nasal and neuralgic affections, and also to the means by which they were finally cured. The pain in the jaw continuing to increase, and from its resemblance to tooth ache, he was induced, September 9th, 1839, to apply to me for advice. From the description which he gave of it and the other circumstances connected with the case, the belief that the antrum was diseased, and that a morbid condition of some one or more of his teeth or their sockets, had been chiefly instrumental in its production, forced itself upon me. With a view of satisfying myself more fully on this point, I gave his mouth a careful examination. His teeth, at least so far as their crowns are concerned, were all free from disease, but the socket of the first left superior molaris, which was that of the sensitive tooth, was considerably wasted—the tooth itself, particularly its outer and posterior surfaces, thickly coated with tartar, slightly loosened and partially protruded from the jaw; whilst the surrounding gum was inflamed and spongy. The tooth having thus, as it would seem, from some cause or other, become obnoxious to the parts within which it was contained, and as it had no antagonist, its removal appeared to constitute the first and principal indication of cure. To this, upon its being advised, he readily submited. The operation was followed by a sudden gush of thin, fetid matter from the antrum, which communicated with the socket of the tooth by an opening sufficiently large to admit of the easy introduction of the end of a small goose quill, and a subsidence of pain. The cause of his complicated malady was now revealed. The roots of the tooth were found to be greatly enlarged by exostosis.—*Ib.*

